# Leprosy in Denmark 1980–2010: a review of 15 cases

**DOI:** 10.1186/s13104-015-1768-6

**Published:** 2016-01-05

**Authors:** Huma Aftab, Susanne D. Nielsen, Ib C. Bygbjerg

**Affiliations:** Department of Infectious Diseases and Rheumatology, Rigshospitalet, Copenhagen, Denmark; Global Health Section, Department of Public Health, University of Copenhagen, Copenhagen, Denmark

**Keywords:** Leprosy cases, Clinical findings, Immunology, Treatment

## Abstract

**Background:**

Leprosy, caused by *Mycobacterium leprae*, is a chronic and progressive granulomatous disease affecting mainly the skin and the peripheral nervous system. If left unrecognized, the infection can lead to permanent nerve damage and disability. The clinical presentation depends on the immune response of the patient and can result in a wide spectrum of symptoms. Leprosy is a rare encounter in Scandinavia but remains endemic in some parts of the world, with some areas reporting an increasing incidence. We performed a retrospective record review of leprosy cases in Denmark from 1980 to 2010 with the purpose of presenting the most common geographical, demographic and clinical findings and to discuss the diagnostic and therapeutic challenges of patients with leprosy.

**Case presentation:**

In total 15 cases were reviewed. The majority (87 %) of leprosy patients in Denmark were born in South- and Southeast Asia, and were presumed to have contracted the infection in their countries of origin. Patients were predominately young males (mean age: 28.6 years). Anaesthetic skin lesion with or without nerve enlargement were the most common clinical presentations (73 %). Immunological leprosy reactions were seen in 40 % of the cases. Diagnoses were based on clinical findings and skin biopsies. Treatment length varied but all patients received multidrug regimens.

**Conclusion:**

Leprosy should be kept in mind when encountering patients with suspicious skin lesions originating from leprosy endemic areas or with history of travel or work in the tropics. Due to the long incubation period with symptoms presenting long after immigration or return, clinicians often do not have the diagnosis in mind. The wide spectrum of symptoms and immunological reactions further complicates the diagnostic process. Treatment of leprosy and the complicated immunological reactions, which frequently accompanies the infection, should be performed in collaboration with a specialist.

**Electronic supplementary material:**

The online version of this article (doi:10.1186/s13104-015-1768-6) contains supplementary material, which is available to authorized users.

## Background

Comparative genomics indicates that leprosy originated in Eastern Africa or the Near East, and was spread by human migration to rest of the world. Leprosy was well-recognized in ancient India and China since 4000 BC with the first known written reference to the disease 600 BC. Leprosy is believed to have arrived in Ireland through trade and commerce with the Far East and travelled to Scandinavia through the Vikings; however, some believe that the crusaders brought the disease to Europe from where it spread to the Americas [1]. Others believe that Alexander the Great’s Greek soldiers introduced it to Europe [2]. By the 12th century leprosy had become a widespread disease in Denmark giving rise to numerous leprosy hospitals/centres, demanding a large number of victims and inflicting a great deal of socioeconomic damage [3]. In Norway the disease raged well into the 19th century giving rise to the world’s first national patient registry. In 1873, driven by epidemiological studies, the causative agent of leprosy was discovered by the Norwegian physician Gerard Armauer Hansen [4], but not until a century later, in 1982, an efficient multidrug therapy (MDT) was recommended by the World Health Organisation (WHO) [5]. The global prevalence rate has declined markedly following implementation of MDT. The WHO ‘elimination of leprosy’ goal is less than 1 per 10,000 persons in a given population receiving MDT. Elimination of leprosy at global level was achieved by the year 2000. At present, leprosy is endemic in countries such as India, Indonesia and Brazil, and these countries account for the bulk of newly detected cases. Thus, in 2013, a total of 180,618 cases were registered, and the prevalence rate was 0.32 in 10,000 worldwide [6]. However, no decline was recorded and a rising incidence in some countries indicate continuous transmission. Some attribute this to the intensified leprosy control and new case finding strategies in endemic regions and not an increasing incidence *per se* [7, 8].

The vast majority of infected individuals never develop clinically detectable symptoms and signs. Age and sex are important risk factors for developing leprosy: adolescents aged 10–19 and persons aged 30 or above are the most susceptible. Adult men are twice as likely to develop the infection as adult women [9]. Although, household contacts of multibacillary (MB) cases have an increased risk of developing leprosy compared to the general population, infection acquired outside the household and subclinical infection is being recognized as an potential route of transmission, sustaining the high new detection rate of leprosy [10–13]; 90–95 % of spouses to infective leprosy patients did not acquire the infection in the pre-antibiotic era. Children of leprosy patients have an increased risk of acquiring the infection compared to the spouse, indicating that genetic similarity to the infected parent makes one more susceptible to the infection [14]. Even though some studies have shown an association between susceptibility to leprosy and certain genes/chromosomal regions, more studies are needed to fully understand the genetics of susceptibility to leprosy [15–17].

Today, in Scandinavia, as in most of Europe, leprosy remains non- autochthonous, brought to the region by immigrants from endemic areas, thus explaining its rarity: In Denmark less than one case per year has been reported during the last 30 years, and the leprosy situation in Denmark was last reviewed more than 20-years-ago [18].

The aim of our study was to give a brief overview of the history and of the current general knowledge on leprosy and conduct a retrospective review of leprosy cases from 1980 to 2010 in Denmark with emphasis on clinical characteristics and therapeutic challenges.

### The causative agent

Leprosy is a chronic granulomatous infection caused by the intracellular acid fast bacilli *Mycobacterium leprae* (*M. leprae*) and is most likely transmitted through droplets from the upper respiratory tract of bacilliferous patients. Although transmission through skin under controlled laboratory conditions has been reported, infection through intact skin is not considered possible. The mycobacterium is difficult to study and has not successfully been cultured in vitro but only in armadillos and footpads of nude mice. The infection has low virulence and a mean incubation period of 4–5 years, but the incubation period may be much longer [19]. Incubation time for paucibacillary (PB) infections is assumed to be shorter than MB.

The bacilli exhibit tropism for histiocytes and Schwann cells, causing anaesthetic skin lesions, and sensorimotor function loss [20], and also for cooler parts of the body, such as eyebrows, earlobes and the nose.

Even though *M. leprae* has been isolated from several environmental and animal reservoirs including armadillos (10), most authors agree on humans being the main reservoir of infection [21].

### The clinico-pathological spectrum

The wide spectrum of clinical presentations reflects the complexity of the immune response towards the causative agent and is graded according to the widely used Ridley–Jopling scheme, which is primarily based on the immunological characteristics. Clinical and histo-pathological characteristics are also considered, and delineated as two stable polar forms ranging from polar tuberculoid (TT) to polar lepromatous leprosy (LL), and in between the unstable borderline forms: borderline tuberculoid (BT), borderline leprosy (BB) and borderline lepromatous leprosy (BL). Thus, tuberculoid leprosy (T) refers to TT and BT while lepromatous leprosy forms (L) to BB, BL and LL. Borderline types are more likely to down- or upgrade through immunological reactions; type 1 reactions [22]. Indeterminate leprosy, the earliest clinically detectable form of the disease is immunologically unstable. Most cases can be cured spontaneously, but some cases may go on to develop one of the determinate forms later. Leprosy may even present with nerve affection as the only symptom: pure neural leprosy.

The clinico-pathological features in leprosy are the result of a varying immune response. Thus, T by mounting a strong cell mediated immune response restricts the infection to a single or few well demarcated, dry (anhydrosis) and scaly anaesthetic skin lesions (macules, plaques) with an elevated margin and central healing. The skin changes are often hypopigmented in dark skin and red/coppery coloured in lighter skin. Although early and significant nerve enlargement is common in T, and the most commonly affected nerves (most common one or few asymmetric nerve affections) are posterior tibial, ulnar, median, lateral popliteal, facial, and radial nerves (may also be affected in L), thickening of the cutaneous nerves close to the skin lesion is an important diagnostic clue.

T lesions are dominated by CD4+ cells, histologically characterized by well-defined granulomas consisting of lymphocytes, epitheloid and giant cells and virtually no bacilli [23], i.e. paucibacillary. The cellular infiltration may extend up to the epidermal layer of the skin. The strong immune reaction in T is probably a result of switching towards a Th1 response. The Th1 type response in patients at the tuberculoid pole (TT and BT) results in abundance of interferon -γ (INF-γ), Tumor Necrosis Factor (TNF-α), interleukins (IL)-2 and IL-15 and a positive skin test (Lepromin test). At the opposite pole (L), the clinical picture reflects a weak cell mediated immune response leading to disseminated infection with multiple skin lesions often distributed symmetrically on the body. In contrast to T, L patients have multiple erythematous or slightly hypopigmented skin lesions (macules, papules and nodules), with poorly defined borders and smooth shiny surfaces which are not necessarily anaesthetic in early stages of the disease. Nerve enlargement and palsy also develop later as compared to T: most common as multiple symmetric nerve affection. Progressive L cases can develop a disseminated infiltration of the skin causing a waxy appearance and thickening of the skin, most prominent in the face, *facies leonidae*. L cases may display systemic symptoms affecting the eyes, testes, bones and liver.

L lesions are characterized by few T lymphocytes with a predominance of CD8+ cells, no granulomas, diffuse infiltration of undifferentiated ‘foamy’ macrophages and loads of bacilli, i.e. multibacillary. The epidermis stays intact until late stages of the disease. In contrast to the immune response seen in T, L lesions formed during the Th2 type response contain transcripts for IL-4, IL-5 and IL-10, are non-reactive to the Lepromin test and show high anti-M. leprae antibody titres which do not seem to convey any protection [24–26].

The unresponsiveness to *M. leprae* extracts in the Lepromin test in L may be explained by immune deviation (the above described Th1 and Th2 immune responses), deletion (varying Th0 cell responsiveness to *M. leprae)* and/or inhibition of T cell responses by regulatory T cells (Tregs) [27]. Some lepromatous patients are capable of mounting a Th1 response upon stimulation with cytokines such as IL-12 secreted by activated dendritic cells and stimulate naïve T cells which are present in T but not L lesions. This observation has led some to conclude that the Th2 immune status in L is reversible [28]. Findings from some studies though do not support the notion of immune deviation in leprosy [29] wherefore increasing interest in clarifying the role of Tregs in leprosy has evolved. However, studies of Tregs in leprosy have found conflicting results as both increased and decreased frequency of Tregs in L patients have been found [30–32]. Thus, more investigations into the role of Tregs in leprosy are needed [33].

### Diagnosing leprosy

According to WHO diagnostic criteria, a person with one of the two cardinal signs: (1) positive skin smear *or* (2) characteristic anaesthetic leprosy skin lesions with *or* without nerve thickening/enlargement with sensory or motor loss, is regarded as having leprosy. WHO recommends a simpler classification scheme based on number of skin lesions and bacillary load in skin smears. This is often used to classify leprosy based on number of skin lesions alone, where Paucibacillary (PB) leprosy corresponds with 1-5 skin lesions (indeterminate leprosy, TT and BT) and MB leprosy with >6 skin lesions (BB, BL and LL) [34].

However, a considerable amount of patients will be misclassified with risk of both over- and undertreatment. Therefore, if not used in the field for operational purposes, experts recommend classifying patients according to the Ridley–Jopling scheme. Thus, whenever possible, a patient suspected of leprosy should undergo examination for the three cardinal signs of leprosy: (1) thorough inspection of the skin for leprosy lesions (morphology described above) and anaesthesia (light touch, pin prick and temperature) of such, (2) enlargement with *or* without sensory or motor loss of most commonly affected peripheral nerves (predilection sites mentioned above), and (3) positive skin smear. A total of 3–6 skin smears should be performed from skin lesions and in L cases from cooler sites with a high probability of finding AFB (earlobes, the forehead, extensor surfaces of the forearms and knees). Skin smears are used to assess the bacillary load: the bacteriological index (BI) and the viability of the mycobacteria; the morphological index (MI). The BI indicating the bacterial load is expressed on a logarithmic scale from 1 + (at least 1 bacillus in every 100 fields) to 6 + (at least 1000 bacilli in every field). Leprosy patients with a BI < 2 are designated as PB while a BI > 2 as MB. Before WHO recommended the current operational method of counting skin lesions to classify a patient as PB or MB, a positive skin slit smear from any site designated the patient as MB. Skin slit smears show poor sensitivity (particularly in PB cases), but their high specificity is very useful for identifying MB, which is the most infectious.

Skin biopsies are usually performed when there is doubt about the diagnosis and remain the golden standard. Biopsies should be taken from an ‘active’ site i.e. red, enlarged and infiltrated. A modified Ziehl-Neelsen stain, such as the Wade Fite stain is preferable for the histological diagnosis. The histopathological characteristics of T and L are described above. According to WHO, only leprosy bacilli which appear as solid acid fast rods are viable while dead leprosy bacilli stain irregularly; this can aid in assessing the treatment effect.

The Lepromin test is analogous to the tuberculin test used in tuberculosis. It relies on the host’s ability to mount a delayed hypersensitivity reaction after an intradermal injection of *M. leprae* antigens. The Lepromin test has low sensitivity and specificity, and a long waiting period of 3 weeks before a reaction can be observed after injection; therefore the test is rarely used. It has no diagnostic value, and was previously used to classify leprosy patients in tuberculoid when positive, and in lepromatous when negative.

Serological tests are under development but are not used routinely due to their low sensitivity in patients at the tuberculoid pole, and have shown false positive reactions in non-leprosy patients [35]. Some studies indicate that antibodies specific *M. leprae* antigens may be useful for detection of early disease in house hold contacts and for monitoring treatment efficacy [36, 37].

PCR for *M. leprae* is sensitive and specific but not widely applied due to cost. Its use until now has been for epidemiological research purposes. In endemic areas it has a promising role for detection of early leprosy, PB cases with a low bacillary load and relapse [38–40].

### Treatment

The WHO MDT for leprosy has proven to be highly effective and should be the first choice for all patients. Patients with up to five skin lesions and/or no bacilli (PB) are treated for 6 months with daily dapsone and monthly rifampicin, while multibacillary (MB) cases with more than six skin lesions and abundant bacilli should undergo a 12 months course of daily dapsone and clofazimine supplemented by monthly rifampicin and clofazimine [41]. Skin lesions in MB patients with a high BI may clear more slowly and have a high BI at the end of treatment compared to MB patients with a lower BI; if such patients do not show improvement after completion of 12 months of MDT, WHO recommends an additional 12 months of MDT.

Severe haemolysis can follow dapsone treatment in patients with Glucose-6-Phosphate Dehydrogenase (G6PD) deficiency. Dapsone should therefore be avoided in these patients. An adverse effect of clofazimine which doctors should be aware of, since it can lead to poor compliance, is reddish discoloration of the skin, conjunctivae and body excretions such as sputum and urine.

Second line agents, ofloxacin and minocycline can be used in a single dose regimen for PB cases; Rifampicin-Ofloxacin-Minocycline, ‘ROM,’ for patients with a single leprosy skin lesion. This regimen was introduced by WHO in 1998 and should only be administered after careful examination of the skin making sure the patient only has one lesion and no nerve involvement. Since ROM has proven to be less effective than MDT for treatment of PB cases with more than one skin lesion, it is currently not recommended for PB cases presenting with 2–5 skin lesions [42].

### Leprosy reactions

Around 30 % of borderline cases experience type 1 [43] reactions resulting in an upgrading of the cellular immune response, shifting the patients towards the tuberculoid pole and a Th1 response. Leprosy type 1 reactions are type IV delayed hypersensitivity immune reactions. The reactions are most commonly observed at initiation of therapy or during the puerperium. However, when the immune system fails to contain the infection a downgrading towards the lepromatous pole is taking place. Distinction between up- and downgrading may be difficult, and may require histo-pathological examination, although downgrading is usually observed prior to initiation of leprosy treatment, whereas upgrading, often occurs as a response to treatment. Type 1 up- and downgrading reactions cause acute inflammation of existing skin (oedema and ulceration) and nerve (motor-sensory loss) disease, new lesions and nerve involvement can also appear. Prompt diagnosis and treatment is of utmost importance due to the risk of permanent nerve damage. Patients should undergo thorough examination of nerves most commonly affected (as described earlier) and followed closely. Prednisolone starting at 30–40 mg tapered to 5 mg over 5–6 months under close observation of nerve function is generally recommended for type 1 reactions; there is however, currently no consensus on dosage and treatment duration [43]. Results are awaited from controlled trials looking into different treatment regimens with prednisolone for leprosy patients with nerve impairment, and the effect of prednisolone prophylaxis for subclinical nerve function impairment [44].

Type 2 reactions, erythema nodosum leprosum (ENL) are mainly seen in BL and LL patients of whom 50 % (the 50 % refers to the subpolar LL group which is immunologically unstable compared to the LL polar form) are at risk of experiencing this complication. ENL is a type III immune response brought about by the inflammatory reaction towards immune-complexes. The immune-complexes deposited in various organ systems cause fever, erythematous painful nodules, uveitis, neuritis, arthritis and orchitis [45]. ENL lesions are characterized by heavy neutrophil infiltration and high levels of TNF-α. The immunological mechanisms underlying the leprosy reactions are not fully understood [46].

Severe ENL can be life threatening and require prolonged immunosuppressive treatment. WHO recommend corticosteroids 1 mg/Kg body weight for 12 weeks. In case of severe ENL not responding to corticosteroid, clofazimine a corticosteroid sparing agents can be used; according to WHO guidelines clofazimine should be started at 100 mg, three times a day for maximum 12 weeks, where after it should be tapered to 100 mg twice a day for 12 weeks and then 100 mg once a day for 12–24 weeks. Clofazimine is more effective than thalidomide for prevention of ENL recurrences. Thalidomide in doses of 100–400 mg/day have shown to be effective in controlling the reaction, but more and larger trials are needed to prove its efficacy [47], and due to its teratogenic side effects, WHO does not recommend its use for the treatment of ENL in women of childbearing age [48]. Despite this: thalidomide is by many practitioners considered to be the most effective drug against ENL. Pentoxifylline is less effective than thalidomide, but has shown to be effective in alleviating ENL symptoms, and could be used as an alternative to thalidomide against ENL when thalidomide is contraindicated [49].

The time frame is important when distinguishing between relapse and leprosy reactions: New elements or nerve affection appearing less than 3 years after a successful course of MDT is more likely to be a reaction. However, the final diagnosis will depend on presence or not of bacilli. According to WHO a relapse in a MB case is defined as “*multiplication of M. leprae, suspected by the marked increase (at least 2*+ *over the previous value) in the BI at any single site, usually with evidence of clinical deterioration”*. To distinguish between relapse and reversal reactions in PB cases a trial of prednisolone is recommended; improvement within 4 weeks favours the diagnosis of a reversal reaction.

Although co-infection with HIV does not seem to shift the patients towards the L pole, some studies indicate an increased incidence of type 1 reactions in patients receiving anti-retroviral treatment, probably as part of an immune reconstitution syndrome [50, 51].

## Methods

Leprosy is a mandatory notifiable disease in Denmark. Based on social security numbers, and retrieved from the national surveillance service database at Statens Serum Institut (SSI) leprosy cases reported in the period January, 1980 to December, 2010 were reviewed. Additionally, we searched the patient register at University Hospital of Copenhagen, Rigshospitalet which is the main referral hospital for tropical diseases in Denmark. For elaboration on medical history, clinical presentation, diagnostic procedures and treatment, medical records, photographs and letters from these and other notifying hospitals were collected.

Permission to obtain information about leprosy cases was granted by the Danish National Board of Health (Additional file 1).

## Case presentation

From 1980 to 2010, 17 cases of leprosy were reported to SSI and five additional cases were identified in the Rigshospitalet patient register. Only 14 of 22 journals were available. One of the cases had been suspected for leprosy, but this was never confirmed. This left us with 13 available medical records of confirmed cases. For two patients, information was collected from letters, photographs and personal correspondence with the attending physician. Thus, in total, information on 15 leprosy patients was reviewed.

The available data were reviewed with focus on demographic data, clinical findings, diagnostic procedures and treatment. The results of these findings are summarized in Table 1.

**Table 1:** Summary of demographic and clinical features of leprosy patients in Denmark recorded from 1980 to 2010

Variable	Leprosy patients (N = 15), n (%)
Age (years)	
Range	5–74
Median	23
Gender	
Male	9 (60)
Female	6 (40)
Country of origin	
Philippines	3 (20)
Cambodia	3 (20)
India	3 (20)
Pakistan	1 (7)
Cameroon	1 (7)
Tanzania	1 (7)
Thailand	1 (7)
Sri Lanka	2 (13)
Duration of stay in Denmark (years)	
Range	0–20
Median	0
0–5	11 (73)
≤6–10	0
≤11–15	0
≤16–20	1 (7)
Unknown	3 (20)
Duration of clinical presentation (weeks)	
Range	3–384
Median	36
Classification	
Tuberculoid leprosy (TT)	5 (33)
Borderline tuberculoid leprosy (BT)/Borderline leprosy (BB)/Borderline lepromatous leprosy (BL)	8 (53)
Lepromatous leprosy (LL)	1 (7)
Indeterminate leprosy	1 (7)
Reactions	
Type 1	4 (27)
Type 2	2 (13)
Histopathological findings	
Skin smears positive for bacilli (7 patients had skin smears performed)	3 (43)
Granulomas in biopsies	9 (60)

Leprosy was predominately imported to Denmark from South- and Southeast Asia (87 %), in particular India, The Philippines and Cambodia, all areas where leprosy is endemic; only two cases originated from African countries. Age of patients ranged from 5 to 74 years, and 11 of 15 patients (73 %) debuted in their second or third decade of life. The youngest patient (5-years-old) was a girl who had most probably contracted the infection from her father, who had been treated for leprosy in Nepal.

Three patients (20 %) recalled exposure to leprosy. Five patients (33 %) were diagnosed and had also undergone treatment for leprosy before migrating to Denmark, while one patient (7 %) was defined as a relapse after consulting a leprologist in London.

Diagnosis was based on travel history, clinical and pathological findings. Most patients were classified according to the Ripley and Jopling scheme. Among the 15 patients 53 % were classified as borderline leprosy types evenly distributed along the spectrum, whilst 33 and 7 % were classified as TT and LL respectively. Duration of symptoms varied from 3 weeks to 8 years, but it was only possible to deduce the length of delay in diagnosis from a few medical records since the date of the first contact with a medical professional was rarely mentioned. For the few for whom the dates were available, the time period from contact with a doctor to suspicion of leprosy was only a few weeks.

Common clinical features were hypopigmentation, slightly dark reddish/brown skin lesions (macules, nodules and plaques), hyposensitivity/anaesthesia of skin lesions, nerve enlargement and palsy, in particular the ulnar and peroneal nerves. Approximately 73 % of cases presented with the two cardinal signs: anaesthetic skin lesions and peripheral nerve thickening. The most common symptom bringing the patient in contact with health authorities was skin changes mimicking conditions such as sarcoidosis and pityriasis versicolor. Initially, two leprosy cases (13 %) were, based on the histo-pathological finding of granulomatous skin changes, mistaken for sarcoidosis.

**Fig. 1:**
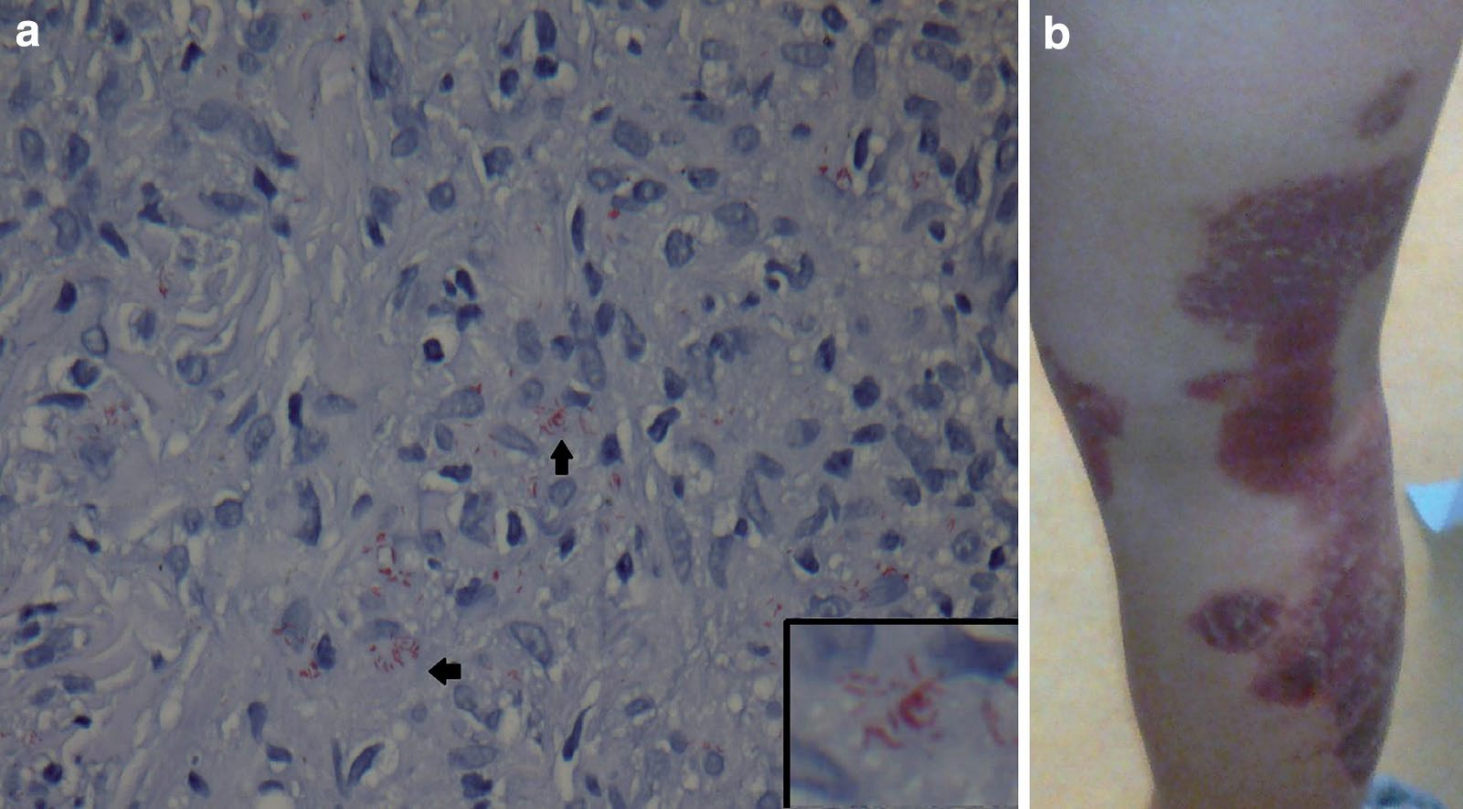
**a** 22-year-old Filipino woman with leprosy initially suspected for sarcoidosis. The patient was designated as a case of BT/BB with a type 1 reaction (**a**). WADE FITE special stain of skin biopsy showing the acid fast *M. leprae* bacilli. **b** *Slightly elevated*, *scaly* and *light coppery coloured plaques* on the *right* leg. Similar lesions were present universally

According to the records available, slit skin smears were performed in seven of the 15 patients (47 %), and the results recorded in six (40 %). The patients from whom skin smears were not obtained were all classified as TT/BT cases. Skin biopsies were obtained from all patients, often on the suspicion of other dermatological disease (Fig. 1). Bacilli were visualized in six of the 15 (40 %) skin biopsies, of which five were borderline leprosy types (BT, BB and BL) while one was a LL patient. Non-necrotizing granulomatous changes were found in nine biopsies (60 %). The Wade Fite stain was used on all biopsies but one. Histopathological description of biopsies from patients with TT was only available for the one patient who experienced a relapse. This showed hyperplasia of the epidermis, perivascular inflammation consisting of lymphohistiocytes, granulocytes and plasma cells and no granulomas. Biopsies from patients in the BT/BB spectrum showed epitheloid cells granulomas, giant cells and an unaffected epidermal zone, while BB/BL was characterized by a more diffuse infiltration with or without lymphocytes, no giant cells and in some cases, ‘foamy’ macrophages and AFB. Confluent infiltrates of histiocytes with neutrophile granulocytes and loads of AFB was the main finding in the LL relapse patient. PCR for detection of *M. leprae* was not performed in any of the 15 cases.

Four of the 15 patients (27 %) experienced a type 1 reaction of which two were classified as borderline lepromatous and diagnosed as downgrading reactions before initiation of leprosy treatment while the remaining presented with type 1 reaction symptoms shortly after initiation of leprosy treatment. The patients with downgrading type 1 reactions displayed the following symptoms: appearance of new anaesthetic skin elements and signs of increased inflammation of the existing ones. Both patients were feverish, one of them with increased C- reactive protein and neutrocytosis. Arthralgia and dactylitis were also present in both cases, while only one of them developed nerve palsy (drop foot). One patient experienced a type 1 reaction shortly after treatment initiation in Cameroun, before arriving in Denmark; the only symptom mentioned in the medical records regarding this was nerve palsy (peroneus paralysis). Half of the patients experiencing a type 1 reaction were treated with corticosteroids.

The patients reacting with ENL (13 %) were BL/LL patients experiencing fever, universal lymphadenitis, flaring of skin elements in the form of inflamed noduli on the upper extremities. One had enlargement of the ulnar and auriuclaris magnus nerves, which was treated with a 1 week thalidomide course while the other received prednisolone due to systemic symptoms with haematuria.

Most common complication/sequelae were decreased sensibility, particularly on the peripheral extremities, ulcers and secondary infections due to neuropathy, as well as keratosis and atrophy of fingers and toes. These patients were referred to an orthopaedist and/or orthopaedic surgeon for evaluation and treatment. Two patients had undergone amputation of fingers/toes, in total grade 2 disability (G2D) was recorded for three patients (20 %). One of three patients with G2D had the amputations done in Cameroun, while another had received several years of leprosy treatment in Thailand, and had been living with G2D for some time when a new reaction (ENL) brought him to a physician in Denmark; bringing G2D to 6 % in our patient sample.

Some patients experienced improvement of their sensory disturbances during treatment but none had complete remission.

Skin lesion with findings of granulomatous changes in skin biopsies often led to the suspicion of sarcoidosis. Other differential diagnoses mentioned were mostly dermatological: acne, pityriasis versicolor, erythema multiforme and Sweets syndrome.

All patients, diagnosed with leprosy after 1982, when WHO launched the use of MDT, received this regimen. Duration of treatment varied and was not for all cases in accordance with WHO recommendations. Some received prolonged MDT (up to 12 years) due to continued presence of bacilli in skin samples, despite clinical improvement.

## Conclusion

Leprosy remains a sporadic disease imported to Denmark. With less than one case detected per year it remains difficult for doctors in non-endemic countries to recognize or even to think of leprosy, which may lead to delay of diagnosis and treatment [52]. Delayed diagnosis may lead to blindness, permanent nerve-damage and other disabilities. Even though anaesthesia of skin lesions is an important distinguishing feature it is often not recognized by the patient.

According to some leprologists there is a predominance of male leprosy patients after puberty, this was also the case in our patient sample. Skin smears were not performed in 53 % of the patients and although these were classified as TT/BT cases not expected to be skin smear positive, skin smears should be performed in all patients suspected for leprosy. Only one patient could recall close contact to an infectious leprosy patient in the household.

Skin biopsies, perhaps because of diagnostic uncertainty, were performed in all patients. Demonstration of acid fast bacilli was not possible in seven of the 15 (60 %) skin biopsies; therefore exposure, travel history and clinical presentation were essential to supplement histology. The more sensitive PCR for *M. leprae* could have proven helpful in these cases [53].

Classification of the disease is important for several reasons: borderline patients are unstable and at risk of experiencing type 1 reaction that can lead to serious nerve damage, while lepromatous and borderline lepromatous leprosy is associated with type 2 reactions which often are complicated by systemic effects. Both these conditions require corticosteroids and close monitoring of the clinical course due to the tendency to recur causing further damage [54, 55]. Another aspect making classification important is when choosing treatment regimen: cf. WHO’s recommendations which are only 6 months for paucibacillary against 12 months for multibacillary leprosy. Skin samples can be positive for non-viable-bacilli years after completion of MDT, and this is not an indication for prolongation of treatment. Unfortunately, viability may be difficult to determine, unless the bacilli are looking disintegrated/discoloured at microscopy. There are no standard international guidelines for how often and how long after completion of MDT a patient should be examined with skin smears. It is also unclear how long a patient should be followed after ending treatment. WHO recommends skin smears at the start of treatment and if relapse/deterioration is suspected. Leading leprologists prefer to discharge patients if they display no complications and are smear negative, except MB patients, who are followed for 2 years after completion of MDT, whilst patients with permanent eye and/or nerve damage are followed lifelong.

Due to the long incubation period, the diagnosis should be kept in mind when encountering patients from endemic areas displaying dermatological or neurological symptoms even years after immigration. Special attention and care should be given to detection and treatment of complications, in particular to those with neuropathy, which can be lasting. Anaesthetic skin lesions, which were the most common clinical finding in our review, should lead to a high suspicion of leprosy.

## Consent

The Danish National Board of Health granted permission to publish this case report. Written informed consent was obtained from the patient for publication of case reports and any accompanying images.

## Additional file

10.1186/s13104-015-1768-6 CARE checklist was followed and used as a guideline for the development of the case report for this manuscript.

## Abbreviations

AFB: acid fast bacilli
BB: borderline leprosy
BL: borderline lepromatous leprosy
BT: borderline tuberculoid leprosy
ENL: erythema nodosum leprosum
G2D: grade 2 disability
IL: interleukin
INF-γ: interferon -γ
LL: lepromatous leprosy
MB: multibacillary
MDT: multidrug therapy
PB: paucibacillary
SSI: Statens Serum Institut
TT: tuberculoid leprosy
TNF-α: Tumor Necrosis Factor
WHO: World Health Organization
